# Early and Mid-Term Outcomes of Coronary Protection during Transcatheter Aortic Valve Replacement: A Single-Center Retrospective Analysis

**DOI:** 10.31083/j.rcm2511407

**Published:** 2024-11-20

**Authors:** Jiawei Zhou, Yuehuan Li, Jinglun Shen, Kaisheng Wu, Jiangang Wang, Yi Yu, Haibo Zhang

**Affiliations:** ^1^Department of Cardiac Surgery, Beijing Anzhen Hospital, Capital Medical University, 100029 Beijing, China; ^2^Department of Cardiology, Beijing Anzhen Hospital, Capital Medical University, 100029 Beijing, China

**Keywords:** transcatheter aortic valve replacement, coronary obstruction, coronary protection

## Abstract

**Background:**

Coronary obstruction (CO) is a fatal complication in transcatheter aortic valve replacement (TAVR). However, data on the outcomes and details of coronary protection (CP) use in TAVR are limited.

**Methods:**

We retrospectively analyzed the patients who had undergone CP during TAVR at our tertiary cardiac center from March 2017 to January 2024. CP was achieved by an undeployed coronary balloon or stent positioned within the coronary artery, which releases the stent at CO occurrence. Patients' computed tomography (CT) evaluation reports and perioperative and follow-up outcomes were reviewed.

**Results:**

A total of 33 out of 493 patients (6.7%) underwent CP during TAVR due to the high risk of CO based on preoperative CT analysis. The mean sinus dimensions measured 30.1 ± 3.6 mm, 29.2 ± 3.4 mm, and 30.4 ± 3.7 mm for the left, right, and non-coronary sinus, respectively. The average left main height was 11.7 mm, and the right coronary height was 14 mm. Self-expanding valves were used in 93.9% of the patients. Coronary balloons were used for CP in 30 patients, whereas undeployed coronary stents were used in three cases. A total of 36 coronary arteries were protected, including 28 left coronary arteries alone, two right coronary arteries alone, and three dual coronary arteries. Eight patients (24.2%) developed CO and underwent stent release. The in-hospital and 30-day all-cause mortality rates were 9.1% and 0%, respectively. The median follow-up time was 10 months, and only one patient died 2 months after discharge due to stroke during the follow-up.

**Conclusions:**

Pre-emptive coronary balloons or stents for CP allow for revascularization in the shortest possible time in the event of CO. Early and mid-term outcomes of CP during TAVR in patients with a high risk of CO show that CP is safe and feasible.

## 1. Introduction

Transcatheter aortic valve replacement (TAVR) is gradually becoming a mainstream treatment for aortic stenosis, even in surgically low-risk patients [1]. Coronary obstruction (CO) is an uncommon (<1%) but life-threatening complication of TAVR, with a 30-day mortality rate of 41% [2]. Many predictors of the development of CO have been reported in the literature, such as being female, previous surgical bioprosthesis, lower-lying coronary ostium, and shallow sinus of Valsalva, etc. [2, 3]. However, detection and prevention of CO in TAVR remain a significant challenge in clinical practice. Because of the high mortality rate of CO, patients at a high risk of CO need appropriate coronary protection (CP). CP with a coronary balloon or stent is a prophylactic technique to manage CO during TAVR [4, 5]. However, detailed procedural analyses and data on the safety and efficacy of this technique in patients at risk of CO are limited. This study aimed to evaluate the early and mid-term outcomes of CP by coronary balloon or stent in patients at a high risk of CO during TAVR.

## 2. Methods

From March 2017 to January 2024, 493 cases of TAVR procedures were performed at our tertiary cardiac center. We retrospectively analyzed the clinical data of the patients. The patients undergoing TAVR who were considered to be at a high risk of CO and underwent pre-emptive coronary balloon or stent protection across the coronary ostia were included in the study. The exclusion criteria were as follows: (1) stent implantation because of disease of the coronary ostia; (2) missing clinical data. This study was approved by the institutional review board (No. 2024115X), and the requirement for informed consent was waived because of the retrospective design.

Data on the patient’s aortic valve annular diameter, sinus diameter, coronary opening height, and leaflet length were obtained from their computed tomography (CT) images. Indications of CP included a coronary artery opening height of less than 10 mm, sinus diameter of less than 30 mm, leaflet length above coronary ostium height, and severe leaflet calcification. The heart team made the joint decision to perform CP during TAVR. Methods of CP included prophylactic intracoronary implantation of balloons or stents. Indications for coronary stent release were (1) coronary angiography leaflets affecting coronary flow, such as the white line sign and thrombolysis in myocardial infarction (TIMI) 0–2 coronary flow; (2) ultrasound or electrocardiogram showing myocardial ischemia with hypotension.

For each case of CP, a 6F guide catheter inserted through the contralateral femoral artery or radial artery was used to engage the left main (LM) or right coronary artery (RCA), and a guiding coronary wire of 0.014 inches was advanced to the distal left anterior descending (LAD) or RCA. A guidezilla guide extension catheter was used to disengage the guiding catheter to prevent catheter-induced coronary ostial damage. Then, an undeployed coronary balloon or stent was positioned within the proximal LAD or RCA. Selective coronary angiography was performed after valve release to assess CO. The stent was rapidly released when the patient met the previously mentioned indications for release. The baseline clinical data of the patients were collected; the anatomical characteristics of the patients were analyzed based on CT images, and the clinical outcomes of the patients were followed up. All clinical outcomes were considered in accordance with the Valve Academic Research Consortium-3 criteria.

Normally distributed continuous data were reported as the mean ± standard deviation (SD), whereas skewed data were reported as the median (range). Categorical variables were summarized as counts (percentages). Continuous variables were compared using Student’s *t*-test. Categorical variables were compared using the chi-square test. Survival analysis was performed using the Kaplan–Meier method. Two-sided *p* values < 0.05 indicate statistical significance. SPSS 26.0 software (IBM, Armonk, NY, USA) was used for statistical analysis.

## 3. Results

A total of 33 out of 493 patients (6.7%) undergoing TAVR underwent CP because of the high risk of CO based on preoperative CT analysis. All of the patients underwent transfemoral TAVR. The mean age was 74.5 years, and 72.7% of the patients were female. Sixteen patients (48.5%) had a previous history of coronary artery disease (CAD). Three patients (9.1%) underwent percutaneous coronary intervention (PCI) before TAVR. Eighteen patients (54.5%) were classified as New York Heart Association (NYHA) functional class III or IV. Ten patients (30.3%) had a bicuspid aortic valve. The baseline clinical characteristics of the study population are shown in Table 1.

**Table 1.** **Baseline clinical characteristics**.

		N = 33
Age (years)		74.5 ± 5.5
Female		24 (72.7%)
BMI (kg/m^2^)		24.6 (5.1)
Diabetes		14 (42.4%)
Hypertension		21 (63.6%)
Atrial fibrillation		6 (18.2%)
CAD		16 (48.5%)
COPD		2 (6.1%)
Prior PCI		3 (9.1%)
Prior stroke		4 (12.1%)
Prior myocardial infarction		2 (6.1%)
NYHA functional class III or IV		18 (54.5%)
STS score (%)		8.2 ± 3.7
Bicuspid		10 (30.3%)
Echocardiographic variables		
	LVEF (%)	61.0 (17.0)
	LV (mm)	47.9 ± 6.7
	Mean aortic gradient (mmHg)	57.2 ± 16.7
	Peak aortic gradient (mmHg)	85.0 (41.0)
	Moderate or severe AI	8 (24.2%)
	SPAP (mmHg)	43.0 (21.0)
	Moderate or severe MI	12 (36.4%)
	Moderate or severe TI	6 (18.2%)

BMI, body mass index; CAD, coronary artery disease; COPD, chronic obstructive pulmonary disease; PCI, percutaneous coronary intervention; NYHA, New York Heart Association; STS, Society of Thoracic Surgeons; LVEF, left ventricular ejection fraction; LV, left ventricle; AI, aortic insufficiency; SPAP, systolic pulmonary artery pressure; MI, mitral insufficiency; TI, tricuspid insufficiency.

Pre-TAVR CT scans were performed and analyzed for 33 patients (Table 2). Fourteen patients (42.4%) had severe leaflet calcification. The average annulus diameter was 24.1 mm. The mean sinus dimensions measured 30.1 ± 3.6 mm, 29.2 ± 3.4 mm, and 30.4 ± 3.7 mm for the left, right, and non-coronary sinus, respectively. The mean sinotubular junction height measured 19.7 ± 2.8 mm. The average LM height was 11.7 mm, and the RCA height was 14 mm. The mean left, and median right coronary leaflet lengths were 14.3 and 13.1 mm, respectively.

**Table 2.** **CT scan data of patients who underwent TAVR with CP**.

		N = 33
Severe leaflet calcification		14 (42.4%)
Annulus perimeter (mm)		75.6 ± 7.2
Annulus diameter (mm)		24.1 ± 2.3
Annulus area (mm^2^)		445.5 ± 86.0
Sinus dimension (mm)		
	Left	30.1 ± 3.6
	Right	29.2 ± 3.4
	Non-coronary	30.4 ± 3.7
	STJ height (mm)	19.7 ± 2.8
	STJ diameter (mm)	26.8 (5.3)
	Left main height (mm)	11.7 ± 2.5
	Right coronary height (mm)	14.0 ± 2.6
Leaflet length (mm)		
	Left	14.3 ± 2.2
	Right	13.1 (2.7)

CT, computed tomography; TAVR, transcatheter aortic valve replacement; CP, coronary protection; STJ, sinotubular junction.

**Fig. 1.**
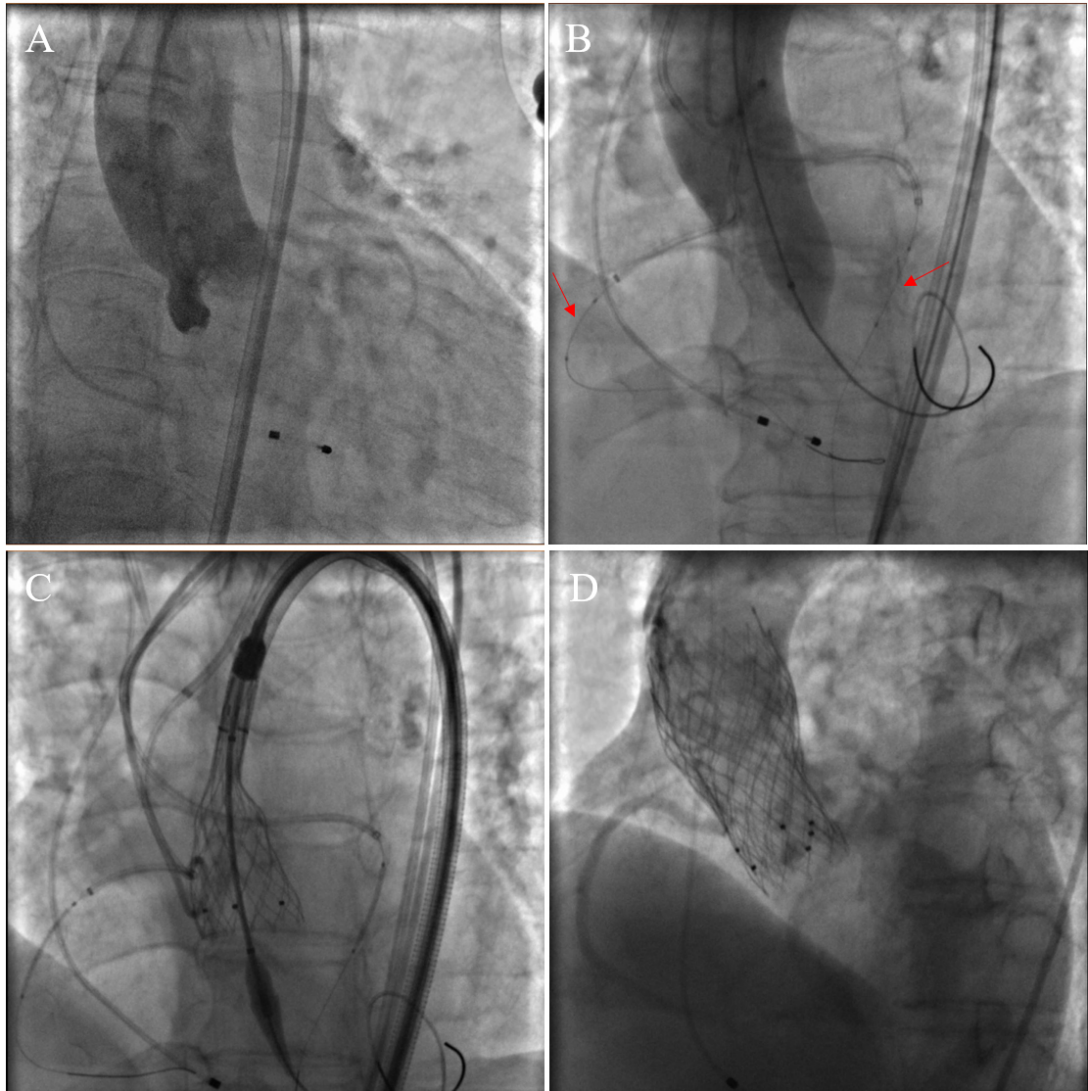
**Procedural images of bilateral CP during TAVR**. (A) Preoperative aortic root angiography. (B) When the balloon was dilated, aortic root angiography was performed to determine the patency of the coronary arteries. Coronary balloons were pre-positioned in both the left and right coronary arteries (red arrows). (C) The self-expanding valve was released gradually. (D) Aortic root angiography after valve release showed patency in the left and right coronary arteries (two valves were used because a paravalvular leak occurred after the first valve was released). CP, coronary protection; TAVR, transcatheter aortic valve replacement.

**Fig. 2.**
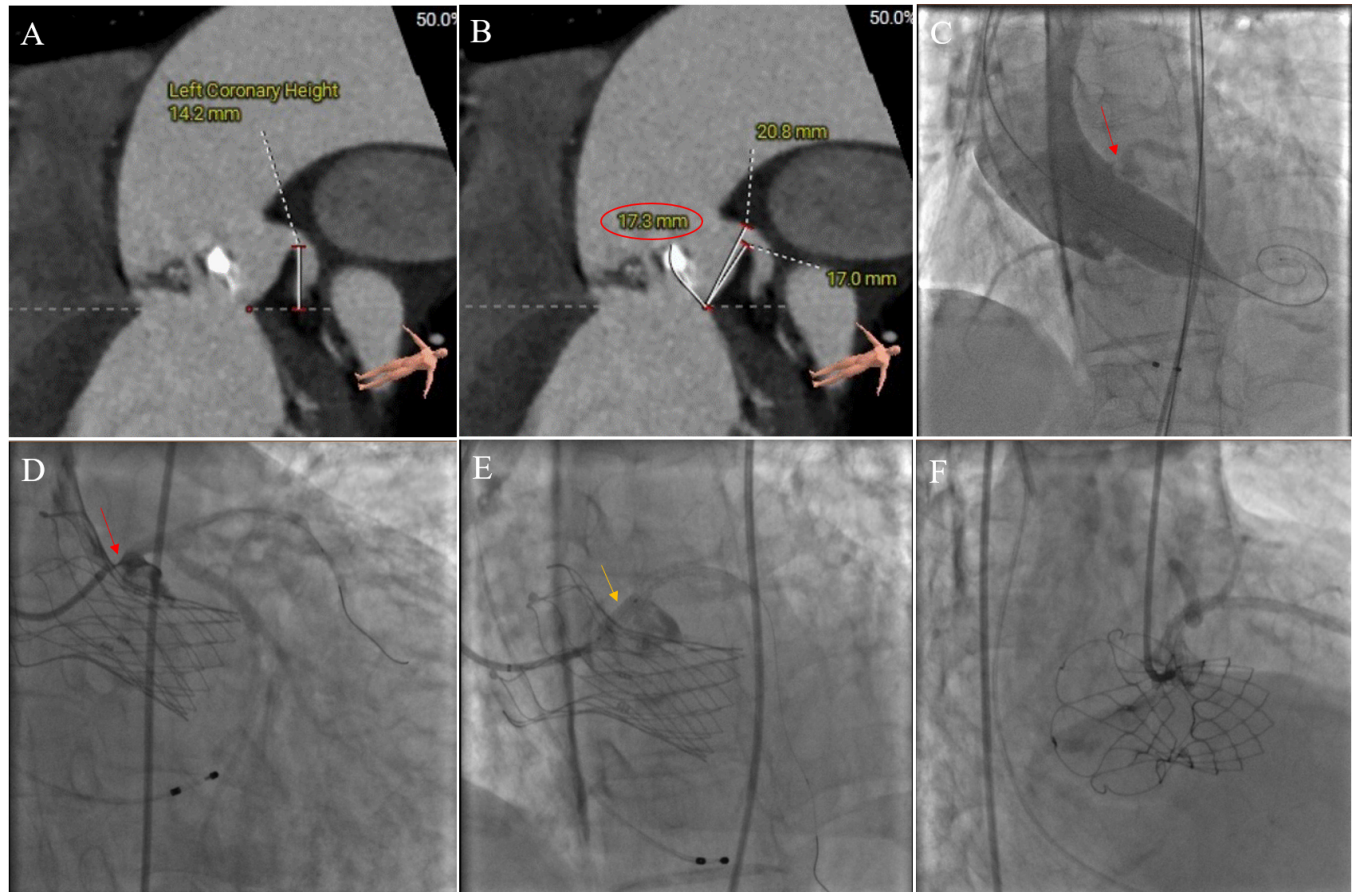
**CO in TAVR without CP was treated with stent implantation**. (A) Preoperative CT analysis showed that the height of the left coronary opening was 14.2 mm, which was not low. (B) The length of the left coronary leaflet was 17.3 mm. The ratio between the leaflet length and curved coronary sinus height was greater than 1. (C) When the balloon was dilated, the native valve leaflet (red arrows) was pushed against the left coronary opening. (D) Localized coronary angiography showed a white line sign (red arrows). Left coronary blood flow was significantly affected by the valve leaflets. (E) The coronary balloon was dilated (yellow arrows), and then a stent was implanted. (F) Final angiogram showing TIMI 3 flow with no stenosis in the left main. CO, coronary obstruction; TAVR, transcatheter aortic valve replacement; CP, coronary protection; CT, computed tomography; TIMI, thrombolysis in myocardial infarction.

**Fig. 3.**
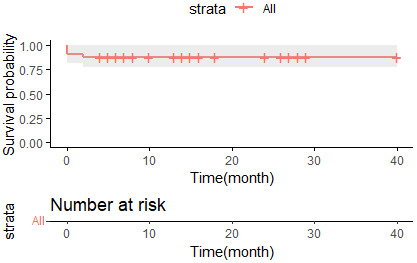
**Kaplan–Meier survival curves for overall survival in TAVR patients receiving CP**. TAVR, transcatheter aortic valve replacement; CP, coronary protection.

Procedural details and outcomes are presented in Table 3. Self-expanding valves were used in 93.9% of the patients. Coronary balloons were used for CP in 30 patients, whereas undeployed coronary stents were used in three patients. A total of 36 coronary arteries were protected, including 28 left coronary arteries alone, two right coronary arteries alone, and three dual coronary arteries (Fig. 1). Thirty-one patients underwent balloon pre-dilation, and six underwent balloon post-dilation. Eight patients (24.2%) developed CO (representative cases are shown in Fig. 2) and underwent stent release. The median length of hospital stay was 4 days. The in-hospital and 30-day all-cause mortality rates were 9.1% and 0%, respectively. Three patients died during hospitalization. Namely, one patient died postoperatively due to low preoperative ejection fraction (30%) and low cardiac output; one patient died due to gastrointestinal bleeding; one patient died postoperatively due to stroke and acute kidney injury. The median follow-up time was 10 months (5.5–18), and only one patient died 2 months after discharge due to stroke during the follow-up. None of the patients experienced a myocardial infarction during the follow-up. The survival analysis of the patients with CP is shown in Fig. 3. The overall survival rate at 3 years was 87.9%.

**Table 3.** **Procedural details and outcomes**.

		N = 33
Valve implanted		
	SEV	31 (93.9%)
	BEV	2 (6.1%)
	Valve size	26 (3)
Coronary protection		
	Coronary protection with balloon	30 (90.9%)
	Coronary protection with stent	3 (9.1%)
Location of coronary protection		
	Left	28 (84.9%)
	Right	2 (6.1%)
	Dual	3 (9.1%)
	Intraoperative PCI	11 (33.3%)
	Intraoperative CPB	5 (15.6%)
	Balloon pre-dilatation	31 (93.9%)
	Balloon post-dilatation	6 (18.2%)
	Coronary obstruction	8 (24.2%)
	Stent deployment	8 (24.2%)
Echocardiographic variables		
	LVEF (%)	60 (9)
	LV (mm)	45.2 ± 6.3
	Peak aortic gradient (mmHg)	17 (10)
	Moderate or severe MI	4 (12.1%)
	Moderate or severe TI	3 (9.1%)
In-hospital outcome		
	Stroke	2 (6.1%)
	PPI	2 (6.1%)
	Perivalvular leakage	4 (12.1%)
	Vascular complication	0
	AKI	2 (6.1%)
	Myocardial infarction	0
	Length of postoperative hospital stay	4 (5)
	In-hospital death	3 (9.1%)
	30-day all-cause death	0 (0%)
	NYHA class I/II	27 (81.9%)

SEV, self-expanding valve; BEV, balloon-expandable valve; PCI, percutaneous coronary intervention; CPB, cardiopulmonary bypass; LVEF, left ventricular ejection fraction; LV, left ventricle; MI, mitral insufficiency; TI, tricuspid insufficiency; PPI, permanent pacemaker implantation; AKI, acute kidney injury; NYHA, New York Heart Association.

## 4. Discussion

The main findings of this study are as follows. (1) In the patients with CP, the left coronary artery was mainly protected (84.8%), while RCA protection and dual coronary protection were less common. (2) Selective coronary angiography at the coronary ostia after valve release revealed the white line sign as an important indication for chimney stent implantation. (3) Early and mid-term outcomes of CP for patients at a high risk of CO during TAVR showed that CP was feasible and safe.

Mechanisms of CO include direct occlusion of the coronary artery ostia or sinus sequestration by the native leaflet [2]. There are differences in the treatment of CO caused by different mechanisms. Low-lying coronary ostium (<12 mm), narrow sinus of Valsalva (<30 mm), a ratio between leaflet length and curved coronary sinus height greater than 1, and short virtual valve-to-coronary distance (VTC, <4 mm) in valve-in-valve (VIV) TAVR have been linked to an increased risk of CO [2, 6, 7]. In our study, low coronary ostium was an important reason for performing CP. The patients in this study had a mean left coronary opening height of 11.7 mm. Leaflet length is another relevant indicator, given that long leaflets may cause CO even in the presence of a high coronary ostium. These indicators can be obtained by preoperative CT analysis, and we can comprehensively determine whether the patient needs CP based on these indicators. Large multicenter clinical studies have shown that the number of cases requiring CP is small, about 2.2% of all cases [8]. However, the percentage of patients who underwent CP in this study was higher, namely 6.7%. The higher rate may reflect differences in the anatomy of the aortic root among different populations.

There are several common approaches to CP during TAVR. The pre-emptive guidewire in the coronary artery: A guidewire is placed in the coronary artery before the release of the transcatheter heart valve (a coronary stent or balloon can also be placed), and the need for further treatment is determined based on the condition of the CO after the valve release. If the coronary angiography is clear and patent, the guidewire can be withdrawn completely without needing protection, although the greatest risk in such patients comes from delayed CO. The advantage of the preventive balloon is that in case of leaflet obstruction, the obstructing leaflet can be pushed away by balloon expansion to restore blood flow quickly and increase time for subsequent management. However, it is necessary to reconstruct the coronary access, which requires time and some experience and skill. The advantage of preventive stent placement is that the operation is relatively simple and can ensure the success rate of coronary opening; however, stent damage or entrapment may occur when withdrawing a stent that does not need to be released. Palmerini *et al*. [8] followed 236 patients at a high risk of CO, and of the 143 patients with a protective coronary stent implanted during TAVR, 93 had a temporary protective coronary guidewire implanted. The results showed that the 3-year cardiac mortality rate was 7.8% for patients in the stent implantation group and 15.7% for patients in the guidewire-only protection group (*p* = 0.05). This suggests that in patients at a high risk of CO during TAVR, prophylactic stenting of the coronary orifice is associated with good mid-term survival. CP with coronary guidewires alone carries the risk of delayed CO.

In a retrospective study that included 60 patients, Mercanti *et al*. [9]stated that chimney stenting as a CO rescue technique can be used to prevent CO, avoiding some adverse events due to hypotension and improving the postoperative benefit for patients. However, because of the difficulty of coronary re-access after chimney stent implantation, it may be indicated only in patients with a very high risk of intraoperative CO and a very low likelihood of future coronary reintervention.

The bioprosthetic or native aortic scallop intentional laceration to prevent iatrogenic coronary artery obstruction technique (BASILICA) is another method to avoid CO. Kitamura *et al*. [10] performed BASILICA in 21 patients proposed for TAVR, with an effective reduction in intraoperative CO risk in 90.5% of patients, with no major vascular complications, need for mechanical circulatory support, stroke, or death within 30 days. Khan *et al*. [11] performed BASILICA in patients at a high risk of CO, with a procedural success rate of 86.9%, the incidence of death and disabling stroke at 30 days was 3.4%, and the 1-year survival rate was 83.9%. Due to the complexity of the technique, we did not use BASILICA. However, we will try it in patients at risk of CO in the future. Interestingly, leaflet-splitting devices have recently emerged to prevent CO [12].

Most of the patients in this study used self-expanding valves mainly because of their recyclability. Such valves can be retrieved immediately when the release process is found to affect coronary flow. Notably, Ahmad *et al*. [13] found that patients with CP using self-expanding valves had a higher risk of CO and a 3-year cardiac mortality than balloon-expandable valves. Yet, this is likely due to their higher-risk clinical and anatomical phenotypes rather than the valve type itself. The higher percentage of LM protection in this study may be due to the lower LM ostium and longer leaflet length; this is consistent with previous reports [2]. Care needs to be taken with an extension catheter and balloon dislodgement from the coronary artery during valve release due to the large number of catheters that are used when performing double CP. Our follow-up findings of favorable early and mid-term outcomes of CP are consistent with other reports in the literature [13, 14].

Indeed, CP increases operative time and puncture access, although, given the severity and unpredictability of CO, it is worth the extra time. Simulations on three dimensional (3D)-printed models can better demonstrate the interaction between the valve and the native leaflets and help predict CO [15]. The white line sign is an important imaging feature for measuring the risk of CO by directly visualizing the native valve leaflet [16]. Meanwhile, intravascular ultrasound is emerging as an important adjunct in detecting CO [17]. All of the patients in this study who had intraoperative stent release took aspirin and clopidogrel postoperatively. No stent stenosis or in-stent thrombosis was found during the follow-up.

## 5. Study Limitations

First, the study was a single-center retrospective analysis with a small sample size. Therefore, larger multicenter, long-term follow-up studies must confirm the results further. Second, there was no imaging or physiological testing to verify the presence of CO. In the future, we will use intravascular ultrasound to aid in diagnosing CO. Third, in most cases, TAVR was performed using a self-expanding valve. Therefore, whether the type of valve results in different outcomes deserves to be explored in the future.

## 6. Conclusions

Pre-emptive coronary balloons or stents for CP allow for revascularization in the event of CO in the shortest possible time. Early and mid-term outcomes of CP during TAVR in patients at a high risk of CO show that CP is safe and feasible.

## Availability of Data and Materials

The raw data supporting the conclusion of this study will be made available by the corresponding authors on reasonable request.
